# Measuring adolescent friendly health services in India: A scoping review of evaluations

**DOI:** 10.1186/s12978-016-0251-8

**Published:** 2016-11-15

**Authors:** Andrea J. Hoopes, Paras Agarwal, Sheana Bull, Venkatraman Chandra-Mouli

**Affiliations:** 1 Department of Pediatrics, University of Colorado School of Medicine, 13123 East 16th Avenue, Box B025, Aurora, CO USA; 2 Department of Community Medicine, Maulana Azad Medical College, New Delhi, India; 3 Department of Community and Behavioral Health, Colorado School of Public Health, 13001 E. 17th Place, B119, Bldg 500, Room E3345A, Aurora, CO 80045 USA; 4 Anschutz Medical Campus, Aurora, CO 80045 USA; 5 Division of Reproductive Health and Research, World Health Organization, Avenue Appia 20, 1211 Geneva 27, Switzerland

**Keywords:** Adolescent friendly health services, Contraception, Adolescent sexual and reproductive health, Reproductive health services, Systematic review, India, Programme evaluation

## Abstract

**Background:**

Initiatives to promote adolescent friendly health services (AFHS) have been taking place in India and many low- and middle-income countries for nearly two decades. Evaluations of these initiatives have been placed in the public arena from time to time, but little is known about what they say about the overall situation on AFHS in India. This study aimed to describe how efforts to provide AFHS in India have been evaluated, how well they have been evaluated, and what their findings and implications are.

**Methods:**

We conducted a scoping review of evaluations of AFHS initiatives in India from 2000 to 2014. An electronic search was carried out in Medline and EMBASE. A manual search of grey literature was also performed, and experts were contacted in order to obtain additional manuscripts and reports.

**Results:**

Thirty evaluation reports were identified representing a broad geographic distribution. Evaluations have focused on government-sponsored AFHS programmes or independent non-governmental organization (NGO) initiatives to strengthen government services. The evaluations primarily measured programme outputs (e.g. quality and service utilization) and health behavioural outcomes (e.g. condom use). Study designs were commonly descriptive or quasi-experimental. Most evaluations found improvement in quality and utilization of services, and some demonstrated an increase in adolescent knowledge or health behaviours. Few measured positive project/programme results such as older age at first pregnancy. Strengths of evaluations were clear objectives, frequent use of multiple data sources, and assessment of programmatic outputs as well as health outcomes. Weaknesses were lack of consistency and quality.

**Conclusions:**

Our findings confirm that a number of evaluations of AFHS initiatives in India have been carried out. They point to service quality and in behavioural improvements in adolescents. However, their lack of consistency hinders comparison across sites, and their uneven quality means that their findings need to be interpreted with caution.

**Electronic supplementary material:**

The online version of this article (doi:10.1186/s12978-016-0251-8) contains supplementary material, which is available to authorized users.

## Plain English summary

Adolescents make up one-fifth of India’s population. India’s government has prioritized efforts to make health services more adolescent friendly. A number of individual studies and evaluations have been carried out and published, but little is known about what they say as a whole. The purpose of our study was to explore the range and results of evaluations of adolescent friendly health services in India.

We conducted a review of publicly-available evaluations of adolescent friendly health service programmes or projects in India from 2000 to 2014. We found thirty evaluations describing initiatives led by government agencies and non-governmental organizations. We summarized the methods and findings of these evaluations using a standard framework. We learned that evaluations were highly variable in measuring programme processes, outputs, or health impacts. Most evaluations found improvement in quality of services and some showed an increased in adolescents’ knowledge and sexual health behaviours.

Our study concluded that evaluations of adolescent friendly initiatives are taking place in India and demonstrating positive health benefits for adolescents. We recommend that evaluation methods be standardized to ensure quality and comparability.

## Background

Improving the reproductive and sexual health (RSH) of adolescents is a key component of India’s National Health Mission [1, 2]. This paper examines evaluations of government and non-government organization (NGO) initiatives to increase access to quality RSH services by adolescents and young people in India.

Adolescents constitute over 20% of India’s population. These young people face a number of RSH problems, such as risk for early and unplanned pregnancy and vulnerability to sexually transmitted infections, including HIV [3, 4]. India’s Ministry of Health and Family Welfare (hereafter called “the Ministry”) addressed these problems in 2005 by formulating its national Adolescent Reproductive and Sexual Health (Adolescent RSH) policy and guidelines within the context of the National Health Mission [5]. Measures were subsequently taken to support their implementation [1]. Officials in some states and union territories began applying the Adolescent RSH policy and guidelines, and NGOs escalated their efforts as well.

A growing body of reports and articles have documented efforts to make RSH services more equitable, available, acceptable, appropriate, and effective-all characteristics of adolescent friendly health services (AFHS) as defined by the World Health Organization (WHO) [6]. In its implementation guide for ARSH, the Ministry enumerated seven standards for providing AFHS (Additional file 1: Table S1) [1]. In 2014, the Ministry launched *Rashtriya Kishor Swasthya Karyakram*, the National Adolescent Health Programme, which expanded the scope of adolescent health programming beyond RSH but maintains AFHS in clinics as a key element of its list of programme components [7]. To date, there is limited knowledge of how these policies and programmes to increase access to quality RSH services have been evaluated and what lessons have been learned thus far.

Our study examined how these expanded efforts to promote AFHS have been evaluated in order to map efforts thus far and identify strategies to perform these evaluations. Specifically, we sought to answer the following questions:

- Where and when have evaluations/studies of AHFS initiatives been carried out?
- Who has conducted these evaluations/studies?
- For what purpose have these evaluations/studies been conducted?
- What design and methods have been used to carry out these evaluations/studies?
- What was the nature and extent of facilities and clients included in these evaluations/studies?
- What were the main findings of these evaluations/studies?

Our goal is to improve the quality and impact of population-based AFHS efforts and to gain knowledge for implementation in other settings.

## Methods

### Literature search

We conducted a systematic search of publicly available peer-reviewed articles and reports from January 1, 2000 to August 1, 2014. We searched Medline and EMBASE electronic databases using medical subject heading (MeSH) terms “adolescent health services” or adolescent and young adult age-limited “health services,” “preventive health services,” or “school health services.” We restricted our search to peer-reviewed studies and evaluations performed in India. Detailed search strategies are in Additional file 1: Appendices 1 and 2. We used the same key words to search websites of organizations engaged in adolescent health service activities in India, including United Nations agencies, international and indigenous NGOs, bilateral agencies, and foundations. In addition, we searched the websites of professional associations and the Ministry at national and state/district levels for relevant publications. Finally, we reviewed the reference lists of articles and reports obtained to identify any additional publications that may have been missed.

### Inclusion and exclusion criteria

**Fig. 1:**
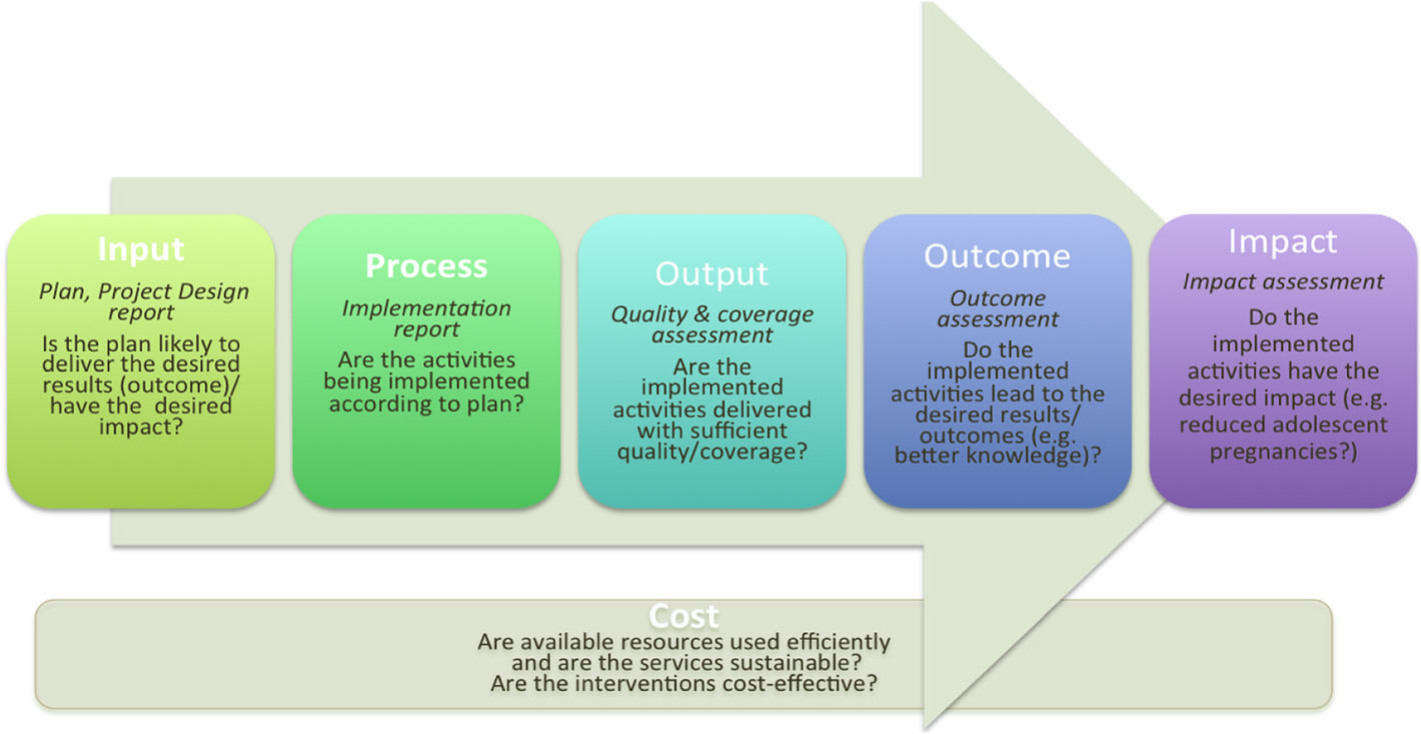
Evaluation logic models

We established inclusion criteria as any report that described an evaluation of an initiative to improve health services for adolescents in India. We included initiatives in all types of health facilities-including those for all ages and those dedicated to adolescents and those operated by government or NGOs. Our primary focus was on facility-based initiatives directed at individuals ten to nineteen years, and on health service provision (i.e. the provision of preventive, curative and rehabilitative services by a trained health worker). We defined evaluation as “the systematic collection of information about the activities, characteristics, and outcomes of programmes [for adolescents] to make judgments about the program, improve program effectiveness, and/or inform decisions about future program development” [7]. We defined research as “the scientific investigation of how social factors, financing systems, organizational structures and processes, health technologies and personal behaviours affect adolescent access to health care, the quality and cost of health care, and health and well-being of adolescent recipients of services” [8]. Because we were primarily interested in results of programmes, we did not include formative or input evaluations that informed programme development and focused our review instead on a range of evaluation types from process to output, outcome and impact evaluations (see Fig. 1). We used standard Preferred Reporting Items for Systematic Reviews and Meta-Analyses (PRISMA) flow diagram to describe the inclusion and exclusion process [9]. PRISMA is an evidence-based flow diagram of the minimum items for reporting in systematic reviews and meta-analyses designed to help authors improve reporting. Human subjects review was not necessary given that our review protocol did not directly involve human participants.

### Data analysis

Two authors reviewed all reports and entered data from those meeting initial inclusion criteria into an evidence table adapted from PRISMA statement elements [10]. We categorized data based on geographic region where the study/evaluation was conducted, year, institution/organization that carried it out, its objectives, its design and methods (see Table 1 for a definition of the types of evaluation or research designs employed across the selected evaluations), nature of health facilities (hospital/clinic, government/non-government), and number of health facilities and/or users studied. We identified the type of study/evaluation they employed organized them into four broad categories (see categories illustrated in the logic model Fig. 1). These categories included findings (when present for each category) specific to process (programme design, fidelity of implementation of the programme), outputs (including quality and coverage/reach of services), health behaviour outcomes, and programme results/impact measured by evaluation. Data entered into the table were discussed with all authors to reach consensus on characteristics and findings of each evaluation. Following data abstraction, we reviewed trends specific to the categories described above and developed primary results for each category through discussions among authors.

**Table 1:** Evaluation or study designs

**Descriptive:** Describes client or programme/project characteristics, service utilization, client satisfaction, and program processes, outputs, and outcomes without a comparison group/site.
**Quasi-experimental:** Compares an intervention group/site to a control group/site without randomization or compares an intervention group/site to itself using measurements pre- and post-implementation of programme/project.
**Experimental:** Compares an intervention group to a control group using randomization.
**Feasibility testing:** Evaluates and analyses the potential of a proposed programme/project.

We utilized the Revised Standards for Quality Improvement Reporting Excellence to assess the quality of each publication [11]. The SQUIRE guidelines were developed and refined through a systematic vetting process with input from an expert panel and through public feedback [12, 13] and provide a framework for reporting new knowledge about how to improve healthcare. Two authors rated each evaluation using an adapted quality assessment scoring approach where each adapted SQUIRE criteria met by an evaluation report resulted in 1 point. A maximum score for meeting all criteria was 15. Two authors independently scored each report, and mean scores and inter-rater reliability were calculated and compared using a Mann–Whitney comparison and kappa statistic.

## Results

We identified 161 publications in our initial database search and thirty-three additional publications from our grey literature search. The process we used to move from this to the thirty presented here is described using a PRISMA flow diagram (Additional file 1: Appendix 3). After removing duplicates, we screened titles and abstracts of 194 publications, of which 141 were excluded. Of the remaining 53 full-text articles and reports reviewed, we excluded twenty-three based on: not examining health service provision (*N =* 14), not specific to adolescents or adolescent-friendly health services (*N =* 5), study/evaluation of programme distributing a health commodity (e.g. iron supplementation) outside of clinical service context/venue (*N =* 3), or other (*N =* 1 non-systematic review). Of the remaining thirty publications, eighteen were published as reports and twelve as peer-reviewed research studies. Characteristics and main findings of evaluation reports (labelled with letters A-S) are found in Additional file 1: Tables S2 and S4 and of peer-reviewed articles (labelled with numbers 1–12) in Additional file 1: Tables S3 and S5, respectively.

### Where and when have the evaluations/studies been carried out?

**Fig. 2:**
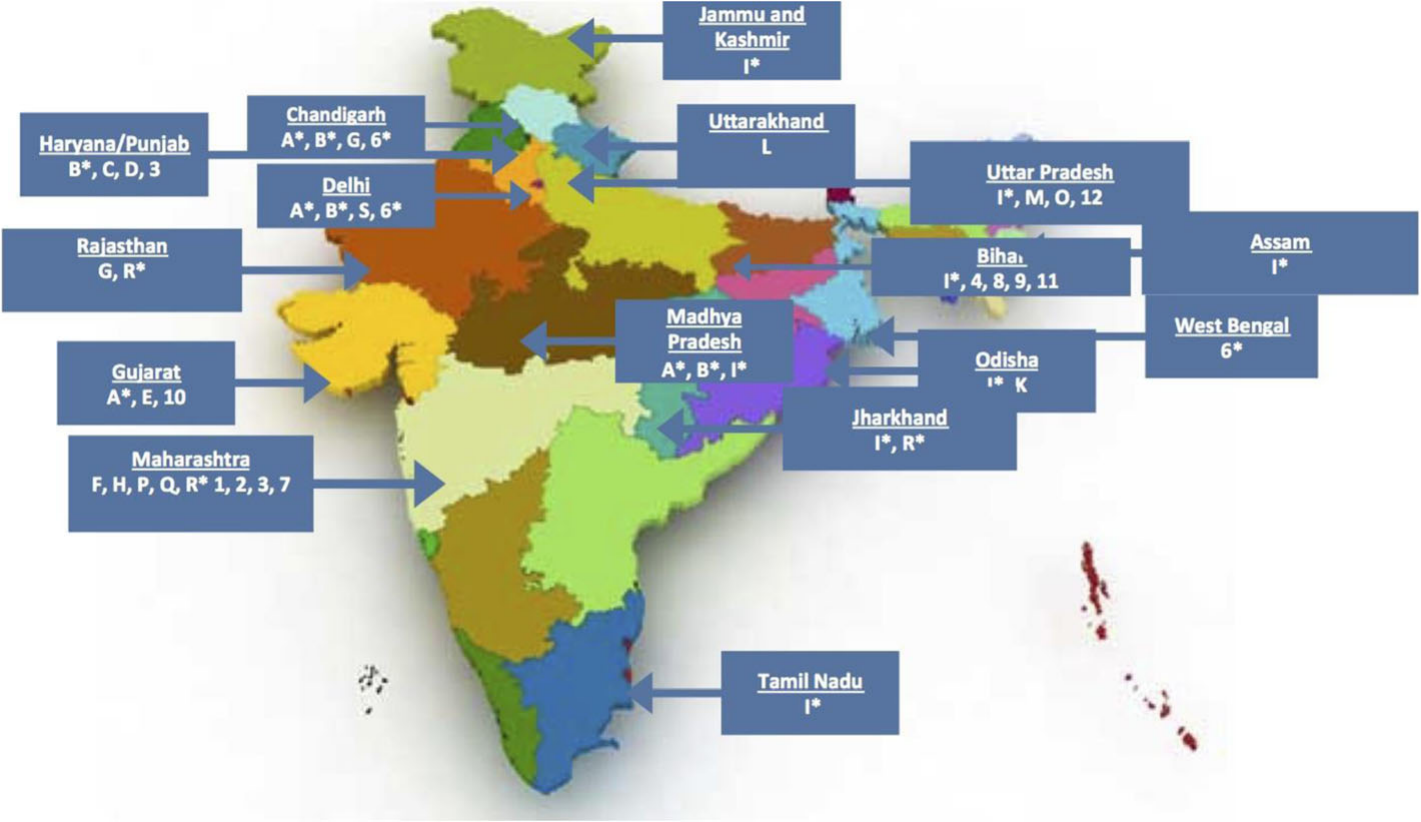
Geographic distribution of evaluations (labelled A through S) and studies (labelled 1 through 12) of adolescent friendly health service initiatives in India

We found a broad geographic distribution of the thirty studies/evaluations. We identified eight in Maharashtra, five in Bihar, three in Haryana, two in Delhi, Gujarat, and Uttar Pradesh, and one each in Odisha, Rajasthan, and Uttarakhand. We also identified five that covered multiple states and union territories. Some evaluations/studies analysed data from the same project (e.g., PRACHAR), at different time points and with varying study designs. See Fig. 2 for a map illustrating where specific evaluations/studies were carried out. The majority of reports/articles were published in the latter half of the inclusion time period of 2000 to 2014 with only five (*A, B; 1,2,3*) published before 2008. Time from AFHS implementation through data collection to publication of report, when indicated, ranged from 1 to 6 years.

### Who has conducted these evaluations/studies?

NGO’s conducted fourteen of the thirty evaluations/studies (46%). Of those, five (*D,M,N; 3,12*) were conducted by indigenous NGOs and nine (*A, B, I, R; 1,5,8,9,11*) by international NGOs. Other bodies included academic institutions (*S, F, K, P, Q, S; 2,4,6,7,10*), consulting agencies (E,G,L), a government (*C*) or a multilateral agency (H). We found many partnerships between NGOs and state government agencies and also that most publications had multiple authors and contributors from different disciplines. The majority of reports/articles (A,B,D,E,F,G,I,L,P,Q,R; 1,3,6,7,9) involved an evaluation/research team that was external to the implementing agency.

### For what purpose have these evaluations/studies been conducted?

Nearly all reports contained clearly defined objectives, often with multiple components. Common objectives were to assess the quality of health services provided to adolescents (*process: C,E,F,G,H,I,K,L,M,N,O,R,S*; *6,10,12),* to assess changes in the utilization of health services by adolescents (*outputs: D,E,H,*K; *2,3,4,12)*, and to measure RSH knowledge of adolescents exposed to a programme (*outcomes: B,J,K,*L; *3,5,9,10).* Few evaluations/studies aimed to assess behavioural outcomes such as condom or contraception use (*outcomes: A, J;4,5*) or health outcomes such as age at first birth associated with programme exposure (*results/impact: 9,11*). One large multi-component project called PRACHAR was evaluated in multiple studies and reports which examined various outcomes including age at first birth, birth spacing, and haemoglobin levels of participants *(J; 5,8,9,11)*. Only the PRACHAR project evaluated the impact on community or population level outcomes such as age at marriage and first birth.

### What evaluation/study designs and methods have been used?

We observed a variety of designs used to perform these evaluations/studies, falling broadly into categories of descriptive, quasi-experimental, feasibility assessment, situation analysis, and those using combinations of designs. A descriptive design was used in most evaluations/studies (*E,F,G,H,I,K,M,O,P,R,S*; *2,4,12*), quasi-experimental in 10 (*A,B,C,D,J,L*; *5,6,8,11*), a feasibility assessment in one (*Q*) and combinations of designs in five (*1,3,7,9,10*).

The most commonly utilized methodology was a simple post-implementation, cross-sectional analysis without a comparison group, found in 18 evaluations/studies (*E,F,G,H,I,K,M,O,P,R; 1,2,3,4,9,10,11*). In contrast, eight (*B,J,L*;1*,3,5,7,10)* applied a pre- and post-implementation (i.e. baseline and follow-up) analysis without comparison groups. We also observed the comparison of “exposed” (facilities/participants who received an AFHS intervention) versus those who were “non-exposed” (facilities/participants who had not received an AFHS intervention): this was used in five evaluations/studies (*A,D;3,6,11*).

In addition to quantitative analytic methods, many evaluations/studies utilized qualitative methods by means of key informant interviews, in-depth client interviews, or focus group discussions to assess various aspects of an AFHS initiative. Qualitative methods were used in 15 evaluations/studies *(E,F,G,M,O,P,R,S; 1,2,3,6,7,9,12).* Details specific to the qualitative analytic techniques were rarely described.

Facility checklists were utilized in a number of evaluations/studies (*C,E,F,G,I,K,L,M,P,S*), and facility attendance records were analysed in five (*2,4,6,7,12*). Provider interviews or questionnaires were used in nine (*E,F,G,K,P,R,S*; 6,10) while adolescent client interviews or questionnaires were used in 12 reports (*A,B,C,F,G,M,O,P,R,S*; *6,12*). One (R) employed mystery clients. Standard definitions of quality varied widely and were inconsistently described in the reports. Only four reports (C,F,Q,P) specifically reported on the seven standards of quality noted in Additional file 1: Table S1 using the quality criteria set out in the Ministry’s implementation guide. (Reference 1), while others (H,K,P,S) describes quality measures that were similar to these standards but not explicitly standardized.

### What was the nature and extent of facilities and service users included in the evaluations/studies?

Where descriptions were provided, there was variability in the nature and extent of health facilities and adolescent users included. Many reports did not contain this information. When information was available, as we found in thirteen evaluations/studies (C*,D,E,F,G,I K,L,M,P Q,R,S*), the size and distribution of target adolescent populations receiving an AFHS intervention was rarely stated. An exception was D, which reports that each cluster of three villages has an estimated adolescent population of 3000–5000, of those approximately 600 adolescents were sampled in each village. Thus, it was often challenging to assess representative nature of a sample or generalizability of the report.

Many reports noted number and kind of health facilities included in the context of a facility assessment (for example, one evaluation in Gujarat (*E*) included twenty-one facilities, representing 50% of all ARSH facilities in the intervention community and one in Rajasthan (*G*) covered 12/110 operating adolescent friendly health clinics (11%), including one of each facility type (district hospital, community health centre, and primary health centre) from each of the four selected districts. From these, evaluators sampled adolescent clients and service providers and also observed facilities using a checklist. Some reports described the number of health service providers or stakeholder interviews, for example, report *E* describes that three state officials, nine district officials, seventeen medical officers, and nineteen grassroots level health workers were interviewed.

We could not infer the representativeness of users surveyed from the information provided. While all evaluations/studies that included surveys or interviews with adolescent clients indicated number of adolescents interviewed, typically stratified by age, rarely did reports describe the sampling population from which these survey participants were drawn or how representative of the sample population they were. Where qualitative methodology was adopted, multiple reports described the number of focus group discussions conducted without indicating the number of participants included in each focus group (*E,M; 9)*.

### What were the main findings of the evaluations/studies?

#### Process

Very few reports commented on process outcomes, specifically programme design or fidelity of programme implementation, and whether any mid-course adaptations were made. The exceptions were report *Q*, which included specific comments about process of designing the programme, and a few which examined feasibility of programmes (*B,Q;1)* or commented on challenges of implementation or monitoring (*E,C,F,G,L)*. Quality was assessed variably across evaluations/studies, with the minority that used the adapted Ministry standards demonstrating an increase across all quality standards compared to control groups or previous time intervals. Persistent unmet quality standards were noted: lack of ensuring adequate equipment and supplies (*P*), inadequate awareness in the community about services (*C,F,Q*) and inadequate management systems in place (*C,F*).

#### Outputs

More evaluations/studies described outputs, with 11 evaluations (*D,E,G,H,M; 1,2,3,4,7,12*) including assessments of service utilization. All but one report (*G*) reported that service utilization increased as a result of an AFHS initiative. However, not all results were presented with baseline data.

#### Health knowledge and behaviour outcomes

In general, programmes designed to make health services more adolescent friendly resulted in increased knowledge about RSH needs of adolescents, both among service users themselves (*A,B,D,L,R,S; 1,3,5,7,10,12*) and among health service providers (*K,10*). Furthermore, a number of evaluations/studies commented on acceptance of the programme by gatekeepers in the community, such as parents (*B,C;1,3*). The most common behaviour outcomes evaluated were self-reported sexual health behaviours, such as condom or contraceptive use (*A,J,L;5,9,11*). In these evaluations/studies, AFHS exposure was associated with increased reported contraceptive and sanitary pad use.

#### Programme results/impact

A small number of initiatives evaluated programme results/impacts such as levels of delayed first birth [9, 11] or anaemia (*B,2*), and an early study (A) of CEDPA Better Life Options Programme examined mean number of children and rates of child deaths, finding both to be decreased. The PRACHAR intervention (*11*) demonstrated greater age at marriage and first birth at the community level.

Using the SQUIRE-adapted scoring system consisting of fifteen questions, the mean quality score averaged between two independent scorers was 8.1/15 (54%). Inter-rater reliability for scores in independent domains was variable (kappa = 0.122, *p =* 0.014), however the average mean quality score was not significantly different (8.53 vs. 7.63, *p =* 0.291).

## Discussion

This is the first study to systematically review a body of country-specific evaluations and studies of AFHS initiatives and to draw conclusions about their quality and their effects. We found that at least 30 independent evaluations and studies have been conducted over a wide geographic distribution of India since 2000. They have been carried out primarily by NGOs and academic institutions and have focused on government-sponsored AFHS programmes or independent NGO initiatives to strengthen government services. The evaluations and studies focused primarily on service utilization trends and health behavioural outcomes and less frequently on design and implementation of AFHS. The rationale for sampling strategies was not uniformly described in evaluation reports, making it challenging to assess the generalizability of the findings. Further, study designs most commonly used were descriptive or quasi-experimental in nature, and frequently lacked a comparison group to draw inferences on effectiveness of initiatives. Future evaluations and studies should be better designed and implemented and should pay more attention to process and long term impact.

Most evaluations/studies demonstrated improvement in the quality of services as a result of government or NGO initiatives to make services more adolescent-friendly. Many also showed an improvement in adolescent knowledge levels of RSH issues, and in health behaviours, such as use of contraception, while few demonstrated positive programme results/impacts.

While much national and international attention has been paid to improving the quality of health systems for adolescents, few efforts to do so have been rigorously studied [14]. It is evident from these evaluation and study reports that a standard approach to evaluation of AFHS has not been adopted. The WHO has developed and promoted the application of its Quality Assessment Guidebook [15] which could facilitate greater comparability across evaluations/studies, but using it will require support —one evaluation (*F*) specifically referenced using WHO quality assessment tools, describing them as “very elaborate and time consuming” and needing to be simplified for local use.

The publication dates reveal that the volume of evaluations and studies of AFHS has increased over time, which is likely attributable to the establishment of the National Health Mission policy and accompanying resources made available for AFHS both by the Government of India and others. Some geographic regions like Maharashtra and Bihar are more represented than others, which may reflect differences in state government support of evaluation resources or external agency interest.

Reviews and syntheses of AFHS in low- and middle-income countries (LMICs) have been conducted at the global level. An example of the former is a review of research and evaluation evidence in improving the quality and use of SRH services by adolescents in LMICs. It found the most robust evidence for programmes that use a combination of approaches including health worker training and facility improvements as well as strategies for demand generation and community acceptance [15]. An example of the latter is synthesis of programmatic outputs (i.e. quality and coverage) and service utilization in eight LMIC countries, which concluded that with support, government-run health facilities can improve the quality of health services and their utilization by adolescents [16].

Moving to measures and methods, a systematic review of indicators of youth-friendly health care in high-, middle-, and low-income countries, identified 22 studies, 15 of which used quantitative methods, six used qualitative methods, and one used mixed methodology [17]. The review further expanded upon eight domains as central to young people’s positive experience of care, including accessibility of health care, staff attitude, communication, medical competency, guideline-driven care, age appropriate environments, youth involvement in health care, and health outcomes. Certain attributes, particularly staff attitudes that were respectful and friendly, were universally applicable while some domains such as clean environment were more dependent to context. While understanding the most appropriate quality indicators is paramount to valuable evaluation, there is little research examining strengths and weakness of different evaluation designs. A recently published post hoc evaluation of a multi-country study on adolescent health provides pointers on good practice in designing and executing studies and evaluations [16]. More attention is needed on the strengths and weakness of different study and evaluation designs on AFHS.

### Limitations

The variety of ways in which evaluations and studies are published and disseminated, ranging from peer-reviewed journals to NGO reports may have limited our ability to access all existing reports. We included only publicly available reports and peer-reviewed journal articles, which may have further limited our access to evaluation reports that have not yet been placed in public domain or may be currently in progress. Further, a publication bias for positive results may have influenced the findings of our review, although our search included reports published outside of the peer-review process. Because the evaluations ranged from brief reports to full evaluation summaries, it is possible that only select findings have been made publicly availably but more thorough evaluation data exists. Furthermore, only few publications provided copies of uniquely developed assessment tools for application in other settings. This presents challenges in comparing evaluation findings across states and also suggests the potential benefit of disseminating validated tools for shared use.

## Conclusions

Evaluations and studies of AFHS initiatives in India are being performed and disseminated. The strengths of these evaluations include clearly stated objectives, frequent use of multiple data sources, and assessment of programmatic outputs as well as health outcomes and impacts. We observed significant variability across study designs in these evaluations, and the target populations and comparison groups were inconsistently defined. Our findings demonstrate that AFHS initiatives have demonstrated improvements in healthcare quality and utilization by adolescents, increased SRH knowledge, and in some settings, improved sexual health behaviours such as condom and contraception use.

India’s new Adolescent Health Programme – *Rashtriya Kishor Swasthya Karyakram* aims to broaden strategies for community-based health promotion and to strengthen preventive, diagnostic, and curative services for adolescents across levels of health facilities [17]. This programme highlights the importance of strong monitoring and evaluation systems, thus it is vital to build upon current knowledge of best evaluation practices in order to ensure the greatest impact to adolescent populations in India and worldwide.

## Additional file

Additional file 1:
**Table S1**. Standards from Government of India Implementation Guide for Assessing Adolescent Friendly Health Services1. **Table S2**. Characteristics of evaluations (N=18). **Table S3**. Characteristics of research studies (N=12). **Table S4**. Main findings of evaluations (N=18). **Table S5**. Main findings of research studies (N=12). **Appendix 1**. Medline search strategy. **Appendix 2**. EMBASE search strategy. **Appendix 3**. PRISMA Diagram. **Appendix 4**. Peer-reviewed publications that were reviewed in full-text and excluded. (DOCX 121 kb)

## Abbreviations

AFHS: Adolescent friendly health services
CEDPA: The center for development and population activities
CORT: Centre for operational research and training
MeSH: Medical subject heading
NGO: Non-governmental organizations
RSH: Reproductive and sexual health
WHO: World health organization
