# Erratum to: Measuring adolescent friendly health services in India: A scoping review of evaluations

**DOI:** 10.1186/s12978-016-0267-0

**Published:** 2017-03-16

**Authors:** Andrea J. Hoopes, Paras Agarwal, Sheana Bull, Venkatraman Chandra-Mouli

**Affiliations:** 1 Department of Pediatrics, University of Colorado School of Medicine, 13123 East 16th Avenue, Box B025, Aurora, CO USA; 2 Department of Community Medicine, Maulana Azad Medical College, New Delhi, India; 3 Department of Community and Behavioral Health, Colorado School of Public Health, 13001 E. 17th Place, B119, Bldg 500, Room E3345A, Aurora, CO 80045 USA; 4 Anschutz Medical Campus, Aurora, CO 80045 USA; 5 Division of Reproductive Health and Research, World Health Organization, Avenue Appia 20, 1211, Geneva, 27 Switzerland

## Erratum

After the publication of this work [1] it was noticed that tables 1–4 and references 17–69 were incorrectly published in Additional File 1 rather than in the manuscript.

The 5 tables and additional references have been added correctly below, and the original version of this article was revised.

The publisher apologises for this error.

**Table 1:** Standards from Government of India Implementation Guide for Adolescent Friendly Health Services ^a^

Standards	Issues covered
1. Availability of specific service package	• Dedicated ARSH clinic (Preventive, Promotive, Curative, and Referral) • Outreach programme for adolescents
2. Delivery of effective services	• Adequate manpower • Guidelines and standard operating procedures • Equipment and supplies
3. Conducive environment at clinic	• Location and timing • Basic amenities • Privacy and confidentiality
4. Sensitive and non-judgemental providers	• Attitude • Communication skills
5. Enabling environment in community	• Sensitization • Distribution of Information Education & Communication (IEC) material
6. Adolescents informed on availability of services	• Signboard • IEC in school, public places • Folk and multimedia
7. MIS in place	• Recording and reporting • Supervision

^a^ (National Rural Health Mission. Implementation guide on RCH II adolescent reproductive sexual health strategy for state and district programme managers [Internet]. 2006. Available from: http://www.searo.who.int/entity/child_adolescent/topics/adolescent_health/rch_asrh_india.pdf

**Table 2:** Evaluation or study designs

**Descriptive:** Describes client or programme/project characteristics, service utilization, client satisfaction, and program processes, outputs, and outcomes without a comparison group/site.
**Quasi-experimental:** Compares an intervention group/site to a control group/site without randomization or compares an intervention group/site to itself using measurements pre- and post-implementation of programme/project.
**Experimental:** Compares an intervention group to a control group using randomization.
**Feasibility testing:** Evaluates and analyses the potential of a proposed programme/project.

**Table 3:** Characteristics of evaluations (N=18)

ID	LOCATION (State: districtblock or villages)	YEAR	ORGANIZATION(S) PERFORMING (“BY”) AND REQUESTING (“FOR”) EVALUATION	OBJECTIVE OF EVALUATION	PROGRAMME EVALUATED	EVALUATION DESIGN	EVALUATION METHODS	FACILITY TYPE EVALUATED	SCOPE OF EVALUATION
**A** [18]	**Delhi:** Periurban slums (district not specified) **Madhya Pradesh:** Indore **Gujarat:** Ahmedabad	2001	BY: Indigenous NGO (Aarogya: Centre for Health-Nutrition Education and Health Promotion based in Fatehganj, Vadodara, Gujarat) FOR: International NGO (The Centre for Development and Population Activities (CEDPA))	To measure behaviour change among participants of a reproductive health promotion initiative (Better Life Options) in areas of education, engagement in incomegenerating activities, decision making mobility, self-esteem/self-confidence, empowerment, fertility, age of marriage, child spacing, use of contraceptives, health seeking behaviour as compared to nonparticipants	Better Life Options Programme components: (1) Building individual capacity through literacy promotion and linkages with formal education (2) Providing family life education (3) Providing vocational skills training (4) Providing age-appropriate general and reproductive health services, (5) Social mobilization through advocacy and community involvement	**Quasi-experimental:** post-implementation comparison of programme participants and non-participants in regards to behavioural and health outcomes	Post-implementation structured interviews with programme participants and nonparticipants using two questionnaires	Type of health facilities within intervention not specified	Number of facilities and adolescent clients using those facilities not specified
**B** [19]	**Delhi:** Slums of South Delhi and East Delhi **Haryana:** Mewat - 5 villages **Madhya** **Pradesh:** 4 unspecified districts	2003	BY: International NGO (CEPDA) and indigenous NGO partners (PRAYATIN in slums of South Delhi, YWCA of India in slums of East Delhi, Society for Promotion of Youth and Masses (SPYM) in slums of Delhi and 5 villages in Haryana, Bhartiya Gramin Mahila Sangh (BGMS) in 4 districts of Madhya Pradesh FOR: International NGO (CEDPA)	To measure the results of the “Adolescent-Friendly Reproductive Health Services Programme” on knowledge and health outcomes of participating adolescents	ENABLE Project: 16 month pilot programme to deliver “Adolescent-Friendly Reproductive Health Services” through 4 NGOs in 3 states of India (Delhi, Haryana, Madhya Pradesh). In addition to traditional Better Life Options programme components (above), ENABLE provided partner organizations opportunity to integrate health services within programme by engaging part-time doctors and lab technicians	**Quasi-experimental:** Pre- and postimplementation comparison of participants’ perception, knowledge and attitudes regarding ARSH issues, further stratified by long-term and short-term intervention-type, and comparison of participants’ haemoglobin levels	(1) Pre- and postimplementation survey assessing perceptions, knowledge, and attitudes (2) Pre- and postimplementation collection of height, weight, and haemoglobin to evaluate effectiveness of adolescent-friendly reproductive health services programme on adolescent female haemoglobin levels	Type of health facilities within intervention not specified	Number of facilities and adolescent clients using those facilities not specified
**C** [20]	**Haryana:** Yamuna Nagar - Kot, Kharwan, Kalanaur, and Burhia blocks	2008	BY: National government agency (Government of India/Ministry of Health and Family Welfare (GoI/MHFW)) FOR: National government agency (Govt of India/Ministry of Health and Family Welfare)	(1) To assess quality of adolescent-friendly health services (AFHS) at selected health facilities in Haryana and to compare quality in AFHS facilities to non- AFHS facilities (2) To determine availability of key health system supports required to implement AFHS (3) To identify barriers to effective implementation of AFHS	Delivering health services based on Government of India’s ARSH Programme	**Quasi-experimental:** post-implementation comparison of ARSH clinics and other clinics in regards to quality indicators of AFHS	(1) Post-implementation interviews of MOs, ANMs, and adolescent clients (2) Assessment of clinics using a checklist	PHCs, CHCs, and SCs offering ARSH	Evaluation covered 10 ARSH clinics and 10 other sites in Both AFHS and non-AFHS sites included 2 PHC and 8 SC evaluations 4 MOs, 16 ANMs, 120 adolescents were interviewed Denominator: ARSH had been implemented in 88 villages served by 4 PHCs, 2 CHCs, and 17 SCs, adolescent population served by facilities not specified
**D** [21]	**Haryana:** Yamuna Nagar	2008	BY: Indigenous NGO (Society for Women and Children’s Health (SWACH)) and state government agency (MHFW), Haryana State) FOR: State government agency (MHFW, Haryana State)	(1) To assess health problems of adolescents (2) To determine baseline data on coverage of key indicators (3) To assess use of SRHS by adolescents in relation to quality of care (4) To assess impact of interventions implemented in selected villages of the district	Delivering health services based on Government of India’s ARSH Programme	**Quasi-experimental:** post-implementation comparison of reported health problems, service utilization, and quality of services among adolescents villages with ARSH versus adolescents in comparison villages without ARSH	Post-implementation household survey of adolescents to measure reported health problems and reported use and quality of SRHS	Type of government facilities not specified	Evaluation covered 30 intervention villages + 30 comparison villages (with 20 adolescents in each) = 599 adolescents from 893 households in intervention villages, 594 adolescents from 868 households in comparison villages Denominator: Each cluster had three contiguous villages with an estimated adolescents population of 3000-5000
**E** [22]	**Gujarat:** District(s) not specificed	2008	BY: Consulting agency (Centre for Operations Research and Training (CORT)) FOR: International NGO (UNFPA) and state government agency (MHFW, Gujarat State)	(1) To evaluate quality of ARSH services (2) To understand utilization pattern of ARSH services and client satisfaction and to analyse factors influencing or impeding service utilization (3) To validate need for special package of ARSH services among adolescents (4) To suggest ways to improve utilization of services and explore possibilities for expanding package of services	Delivering health services based on Government of India’s ARSH Programme	**Descriptive:** postimplementation crosssectional evaluation	(1) Qualitative individual interviews with health workers and government health officials (2) Focus group discussions with adolescent boys and girls (3) Assessment of clinics using a checklist	Type of health facilities not specified	21 facilities visited, of which 17 (81%) were functional and able to be assessed 3 state officials, 9 district officials, 17 MOs, 19 grassroots level health workers 28 focus group discussions with adolescent boys and girls Denominator: 42 total ARSH facilities = 50% coverage; adolescent population served by facilities not specified)
**F** [23]	**Maharashtra:** Raigad-Karjat block	2009	BY: Academic institution/university (National Institute for Research in Reproductive Health (NIRRH)) FOR: National government agency (GoI/MHFW)	(1) To assess status of ARSH services (2) To generate baseline data for identifying gaps in delivery of ARSH services (3) To provide recommendations for improving quality assessment tools	Delivering health services based on Government of India’s ARSH Programme	**Descriptive:** postimplementation crosssectional evaluation	(1) Qualitative interviews with MOs, ANMs, adolescent clients (2) Assessment of clinics using a checklist	PHCs, SCs, and sub-divisional hospital (SDH)	Interviews with 6 MOs, 11 ANMs, 24 adolescent clients Assessment of 10 health facilities (3 PHCs, 6 SCs, 1 SDH)
**G** [24]	**Rajasthan:** Bhilwara, Chittorgarh, Alwar and Kaurali	2010	BY: Consulting agency (India Institute of Health Management Research (IHMR)) FOR: Multilateral agency (UNFPA, Rajasthan State Office)	(1) To assess status of ARSH services in 4 districts in Rajasthan (2) To assess status of training of service providers in ARSH services (3) To assess availability of ARSH information for adolescents (4) To assess preparedness to improve and sustain provision of services	Delivering health services based on Government of India’s ARSH Programme	**Descriptive:** postimplementation crosssectional evaluation	(1) Interviews with health service providers and adolescent clients (2) Assessment of clinics using a checklist	Primary health care centers (PHCs), community health centers (CHCs), and district hospitals (DHs)	Evaluation covered 12 AFHCs in 4 selected districts provided at 1 of each facility type (DH, CHC, and PHC) in each district 24 providers were interviewed 131 adolescents interviewed Denominator: 110 operating AFHCs in 4 selected districts among 8 districts where service package has been implemented. Adolescent population served by facilities not specified
**H** [25]	**Maharashtra:** 33 districts not specified	2011	BY: Multilateral agency (UNFPA) FOR: Multilateral agency (UNFPA) on behalf of multiple state governments throughout India (including Government of Maharashtra for this particular portion of report)	(1) To evaluate the functioning of the AFHCs (2) To assess service environment, status of training of service providers, and availability of information to adolescents with regard to ARSH services	Delivering health services based on Government of India’s ARSH Programme	**Descriptive:** postimplementation crosssectional study of quality of services	Specific methodology not specified	Type of health facilities within intervention not specified	Number of facilities and adolescent clients using those facilities not specified
**I** [26]	**Uttar Pradesh Madhaya Pradesh Jharkand Orissa Assam Jammu and Kashmir Tamil Nadu** (Districts not specified)	2011	BY: Academic institution/university (Population Research Centre, Institute of Economic Growth) FOR: National government (Programme Evaluation Organisation Planning Commission/Government of India)	To evaluate and assess availability, adequacy and utilization of AFHS in rural areas	Delivering health services based on Government of India’s ARSH Programme	**Descriptive:** postimplementation crosssectional study of quality of services	(1) Household survey (2) Facility survey	DHs, CHCs, PHCs, SCs and 296 villages over 37 districts in 7 states	Facility survey covered 37 DHs, 74 CHCs, 148 PHCs, 296 SCs, and 296 villages stretched over 37 districts over 7 states of India 25 households for the household survey in each selected village was based on identification of 5 households under each of the following categories: those having pregnant woman, having lactating women, with children 1-5 years, with at least one chronic disease patient, and having utilized family planning services = 7400 households Denominator: Total number of facilities and adolescent population served by these facilities not specified
**J** [27]	**Bihar**: Nalanda, Nawada, Patna	2011	BY: International NGO (Pathfinder International) FOR: International NGO (Pathfinder International)	To evaluate knowledge, attitude, and practice changes after Phase I and II of PRACHAR intervention as well as impact of PRACHAR IRH training Evaluation specifically looks at differences in impact based on different components of the intervention	PRACHAR intervention: (1) Social environment building (2) Providing info on RH and services (3) Improving access to RH services: training formal and informal rural health service providers on RH issues and contraception, encouraging vulnerable populations to seek services, motivating chemists and village convenience shops to keep regular stocks of condoms and pills	**Quasi-experimental**: Pre- and postimplementation comparison of participants’ contraception attitudes, knowledge, demand, and use	Pre- and postimplementation survey of participants	Type of health facilities within intervention not specified	Health facilities in intervention communities and number of adolescent participants using facilities not specified
**K** [28]	**Orissa**: Kalahandi-Junargarh and Dharmagarh blocks, Rayagada-Rayagada and Gunupur blocks	2012	BY: Academic institution/university (India Council of Medical Research (ICMR)) FOR: ICMR	(1) To assess knowledge, attitude, and behaviour on reproductive health problems in adolescents (2) To assess quality of care at AFHCs (3) To assess accessibility and utilization of health care services by adolescents	Delivering health services based on Government of India’s Adolescent Reproductive and Sexual Health Programme	**Descriptive**: postimplementation crosssectional study of quality of services	(1) Community-based survey of adolescents with measurement of height, weight, midupper arm circumference, haemoglobin of adolescent clients (2) Survey of stakeholders (community health workers school teachers) using questionnaires (2) Facility-based survey of providers (3) Assessment of clinics using a checklist	Adolescent friendly health clinics (type of facility not further specified)	Community sample in 2 districts included 720 households in Kalahandi, 657 households in Rayagada -Covered 858 (Junagarh 567, Dharmagarh 291 in Kalahandi) and 755 (Rayagada 420, Gunupur 335 in Rayagada) adolescents respectively 224 stakeholders interviewed (116 in Kalahandi and 108 in Rayagada) 73 health service providers interviewed (30 in Kalahandi, 43 in Rayagada) Quality of care evaluated at 2 AFHCs in Kalahandi and 1 in Rayagada Denominator: Total number of facilities and adolescent population served by these facilities not specified
**L** [29]	**Uttarkhand**: District not specified	2012	BY: Consulting agency (Futures Group International) FOR: Foreign government agency (USAID)	To compile a summary of numerous published and unpublished materials to capture best practices, lessons learned and recommendations developed over course of 2 years of work on ARSH within Innovation in Family Planning Services (IFPS) Projects and IFPS Technical Assistance Project (ITAP)	UDAAN intervention: (1) Pilot phase: advocacy workshops, recruitment and training of health care providers, peer group educators to work within school-going and out-of-school adolescents (2) Scale up phase: Facilities oriented to become more youthfriendly, establishment of adolescent groups	**Quasi-experimental**: Pre- and midintervention comparison of sexual and reproductive health knowledge and attitudes	(1) Questionnaire measuring SRH knowledge and behaviors among adolescents (2) Unspecified facility assessment tools	Type of health facilities within intervention not specified	80 primary sampling units (PSUs) were selected by sampling 10 villages from 8 pilot blocks. 32 adolescents were selected from each PSU to include 2500 adolescents total in assessment Midterm assessment included 317 adolescents who had used at least one UDAAN services and 1273 who had not used any UDAAN service Denominator: Health facilities in intervention communities and number of adolescent participants using facilities not assessed
**M** [30]	**Uttar Pradesh**: Varanasi-Arajiline block, Bangalore-Hoskote block	2013	BY: Indigenous NGO (Research Unit at MAMTA-Health Institute for Mother and Child, Delhi) FOR: Not specified	To assess youth friendly health services from clients’ perspectives and role of outreach activities in improving access to the services for purpose of potential upscaling	Delivering health services based on Government of India’s ARSH Programme and community outreach through provision of Youth Information Centers (YIC)	**Descriptive**: postimplementation crosssectional study of quality of services	(1) Semi-structured interviews to measure demographics, time spent on client-provider interactions, perception regarding privacy and confidentiality, awareness about YIC activities, role of YIC, level of satisfaction (2) Focus group discussions to measure privacy-confidentiality, attitude of adults towards adolescent concerns, roles of outreach activities in improving access to services (3) Assessment of clinics using a checklist	Youth friendly health facilities not further specified	Consecutive sample of 120 clients from 4 selected clinics for exit interviews 8 focus group discussions (8-10 participants each) conducted among community members and young people Denominator: Total number of facilities and adolescent population served by these facilities not specified
**O** [31]	**Uttar Pradesh**: Hardoi and Siddharth Nagar **Bihar**: Nalanda and Vaishali	2013	BY: Indigenous NGO (Research Unit at MAMTA-Health Institute for Mother and Child, Delhi) FOR: Not specified	To analyse key determinants of YFHS that influence client’s satisfaction level in order to help decision makers implement programmes tailored to clients’ perceived needs	Delivering health services based on Government of India’s Adolescent Reproductive and Sexual Health Programme	**Descriptive**: postimplementation crosssectional study of quality of services	Semi-structured interviews with clients to measure demographics, time spent on client-provider interactions, perception regarding privacy and confidentiality, level of satisfaction	Youth friendly health facilities not further specified	Consecutive sample of 120 clients from 4 selected clinics for exit interviews Denominator: Total number of facilities and adolescent population served by these facilities not specified
**P** [32]	**Maharashtra**: Raigad - Karjat block	2014	BY: academic institution/university (National Institute for Research in Reproductive Health (NIRRH/Indian Council of Medical Research (ICMR) FOR: State government (Government of Maharashtra)	To assess the quality of adolescent health related services in Maharashtra against the 7 ARSH standards established by GoI in 2005	Delivering health services based on Government of India’s ARSH Programme	**Descriptive**: postimplementation crosssectional study of quality of services **Quasi-experimental**: Time series comparison of health service utilization	(1) Structured interview questionnaires for staff and clients (2) Assessment of clinics using a checklist	SDH, PHCs, SCs	10 health facilities: 1 SDH, 3 PHCs, 6 SCs during first year SCs excluded during 2nd year 3 additional PHCs and RH added for 2nd-5th years for total 8 facilities (1 SDH, 6 PHCs, 1 RH) 1 Taluk Health Officer, MO, and ANM interviewed at each site Denominator: Total number of facilities and adolescent population served by these facilities not specified. Number of clients interviewed not reported.
**Q** [33]	**Maharashtra**: Raigad –Karjat block	2014	BY academic institution/university (National Institute for Research in Reproductive Health (NIRRH)/Indian Council of Medical Research (ICMR) FOR: multilateral agency (WHO) and state government (Government of Maharashtra)	To test (in one block of one district) the feasibility of a developed action plan designed to link ARSH and HIV services in two districts.	Linking Government of India’s Adolescent Reproductive and Sexual Health Programme and HIV services	**Feasibility testing**: Observations on implementation of linking ARSH services and HIV services	Did not specify tools for testing feasibility interventions to link ARSH-HIV services	SDH, RH, PHCs,	8 facilities included in evaluation: 1 SDH, 1 RH, and 6 PHCs Denominator: Total number of facilities and adolescent population served by these facilities not specified.
**R** [34]	**Jharkand**: Jamtara and Palamu districts **Maharashtra**: Chandrapur and Nashik; **Rajasthan**: Bhilwara and Karauli districts	2014	BY: International NGO (Population Council) FOR: National government (Government of India/Ministry of Health and Family Welfare)	To identify approaches to enhanced service delivery through adolescent-friendly health centers through refinements in content of and approaches to training and to inform strategies to generate demand for services	Delivering health services based on Government of India’s ARSH Programme	**Descriptive**: Postimplementation mixedmethods cross sectional study	(1) In-depth interviews with ASHAs, ANMS, counsellors, and medical officers (2) Observation of service delivery at AFHCs using mystery clients (2) Exit interviews with clients accessing services (4) Cross-sectional, community-based survey of adolescents	Adolescent friendly health centers in community health centers (CHCs), subdistrict hospital (SDH), or rural hospitals	12 AHFCs were evaluated of total 180 AFHCs in Jharkhand, 140 AFHCs in Maharashtra, and unspecific number in Rajasthan 24 mystery client visits (8 each in Jharkhand, Maharashtra, and Rajasthan) Exit interviews performed with 5 adolescents (4 in Jharkhand and 1 in Maharashtra) Community-based survey covered a proportional distribution of 2131 adolescents from 48 villages within the 3 states (736 from Jharkand, 682 from Maharashtra, and 713 from Rajasthan) Denominator: Total number of facilities and adolescent population served by these facilities not specified
**S** [35]	**Delhi**: All 9 districts	2013	BY: academic institution/university (Maulana Azad Medical College) and state government (Directorate of Family Welfare) FOR: Not specified	To evaluate availability, type and quality of facilities providing RH services to adolescents in public and private sector	Delivering health services based on Government of India’s Adolescent Reproductive and Sexual Health Programme	**Descriptive**: postimplementation crosssectional study of quality of services	(1) Semi-structured interviews with facility managers (2) Facility checklists (3) Questionnaires for service providers at primary, secondary, and tertiary health centers (4) Exit interviews with clients accessing services	Primary, secondary, and tertiary health centres	9 of 9 total district head quarters assessed for availability of services 4 of 9 total districts sample for quality of services: 39 of 39 total facility managers, 31 of 31 secondary and tertiary units, 70 of 250 primary units, and 936 of 907,710 adolescents

**Table 4:** Characteristics of research studies (*N* = 12)

ID	Location (State:District-Block or villages)	Year	Organization(s) performing (“BY”) and requesting (“FOR”) study	Objective of study	Programme mestudied	Study design	Study methods	Facility type studied	Scope of study
**1** [36]	Maharashtra: Dhamari village, Pune	2006	BY: International NGO (ICRW) and academic institution/university (KEM Hospital) FOR: International NGO (ICRW)	To test feasibility in rural context to provide married youth with integrated package of reproductive health care and counselling	Providing integrated package of: (1) Reproductive health information (2) Clinical referrals (3) Reproductive and sexual health couples counselling	**Multiple designs:** **(1) Feasibility assessment:** Observations on implementation of package of reproductive health education, care and counselling for rural married youth. (2) **Descriptive:** Post-intervention cross-sectional study of attendance levels of participants, focus group discussions and interviews with participants (3) **Quasi-experimental:** Pre- and post-test of couples’ RSH knowledge	(1) Methods not specified (2) Analysis of attendance records and referrals made, and follow-up visits that occurred, evaluations (self and external) of community level educators, group discussions and individual interviews with participants (3) Pre- and post-implementation surveys to test couples’ RSH knowledge	Type of health facilities within intervention not specified	Number of facilities and adolescent clients using those facilities not specified
**2** [37]	Maharashtra: Urban Mumbai	2006	BY: academic institution/university (National Institute for Research in Reproductive Health (NIRRH)) FOR: Not specified	To assess the reproductive health problems and help-seeking behaviour among urban school-going adolescents [in context of ongoing intervention in schools in urban Mumbai during 2003-04 aimed at creating model for school-based adolescent friendly services through Adolescent Friendly Center (AFC)]	Providing school-based adolescent friendly services through AFC (established on school premises, function 2 days/week for 2 hours/day; services include information provision, counselling, free medical exams, anonymous letterbox)	**Descriptive:** post-intervention cross-sectional study of students using adolescent friendly center and of clinic attendance trends	(1) Self-administered questionnaire and collection of biologic health data during camp (2) Focus group discussions with male and female students (3) Monitoring of attendance data from clinic	Outpatient clinic on a school premises	300 urban school-going adolescents participated (11-14 year olds) from a single outpatient clinic on a school premises. A separate evaluation (not included) was done for 300 15-19 year olds Details of school population from which this group was sampled not specified
**3** [38]	Maharashtra: 2 unspecified blocks in Ahmednagar	2006	BY: Indigenous NGO (Foundation for Research in Health Systems (FRHS)) and international NGO (International Center for Research on Women (ICRW)) FOR: Indigenous NGO (FRHS) and international NGO (ICRW)	To assess the effectiveness of social mobilization and health services strengthening to improve married adolescents’ reproductive and sexual health knowledge and to increase their access to and use of health services.	(1) Social mobilization strategy implemented through indigenous, community-based women’s and youth organizations to provide structured, interaction and recurrent health education sessions on select reproductive health topics. (2) Strengthening health services was done by working with state government to address specific gaps in training local health officials	**Multiple designs:** (1) **Experimental:** Communities were randomly assigned to social mobilization, strengthening of health services, both or neither and knowledge and utilization of services were compared between 4 arms **(2) Descriptive:** Post-implementation cross-sectional study of husbands in one study arm (3) **Quasi-experimental:** pre- and post-implementation qualitative comparison of mothers-in-law in one study arm	(1) Pre- and post-surveys of young married womens’ assessing knowledge and reported utilization of services (2) Post-implementation survey of husbands of young married women in social mobilization arm involvement and awareness in womens’ reproductive health (3) Pre- and post- interviews of mothers-in-law in social mobilization arm assessing involvement and awareness in womens’ reproductive health	Type of health facilities within intervention not specified (Social mobilization activities involved indigenous, community-based women’s and youth organizations with some district health staff, and health service strengthening involved training of local health officials)	Number of facilities and adolescent clients using those facilities not specified
**4** [39]	Haryana/Punjab: Sectors 19 and 38 of Chandigarh City	2008	BY: academic institution/university (Post Graduate Institute of Medical Education and Research) FOR: Not specified	(1) To assess perceived health problems and help seeking behaviour of adolescents (2) To measure utilization of adolescent health clinics by adolescents	Establishing adolescent health clinics in two diverse settings; a school-based clinic and a dispensary-based clinic	**Descriptive:** post-implementation cross-sectional study of self-reported health problems and help-seeking behaviours and of pattern of service utilization of clinic over preceding year	Semi-structured questionnaire and analysis of clinic utilization records	School-based clinic and dispensary-based clinic, both described as “adolescent health clinics”)	360 adolescents using 2 facilities (1 school-based clinic and 1 dispensary-based clinic) were selected by stratified random sampling from a population of 3000 adolescents (2100 from sector 19 in 2 schools and 900 from sector 19 in 1 school)
**5** [40]	Bihar: Nalanda, Nawada, Patna	2008	BY: International NGO (Pathfinder International) FOR: International NGO (Pathfinder International)	To assess effect of PRACHAR intervention on: (1) contraceptive demand and use and (2) related attitudes and knowledge	PRACHAR intervention: (1) Social environment building (2) Providing info on RH and services (3) Improving access to RH services: training formal and informal rural health service providers on RH issues and contraception, encouraging vulnerable populations to seek services, motivating chemists and village convenience shops to keep regular stocks of condoms and pills	**Quasi-experimental:** Pre- and post-implementation comparison of participants’ contraception attitudes, knowledge, demand, and use	Pre- and post-implementation questionnaire	Type of health facilities within intervention not specified	Health facilities in intervention communities and number of adolescent participants using facilities not assessed
**6** [41]	Delhi, West Bengal, and Chandigarh: South West Delhi District, Chandigarh- Sector 32, Kolkata District of West Bengal state	2009	BY: Academic institution/university (India Council of Medical Research (ICMR)) FOR: National government agency (Ministry of Health and Family Welfare (MHFW) and multilateral agency (World Health Organization)	To examine whether adolescent friendly health centres (AFC) have increased the quality and access to health services as per the client’s perception	Delivering health services based on Government of India’s Adolescent Reproductive and Sexual Health Programme	**Quasi-experimental design:** Comparison of quality and utilization of ARSH with corresponding “control” outpatient clinics	(1) Interviews with key stakeholders (staff members, adolescents, parents) (2) Review of relevant documents (not clear if a facility assessment was performed)	ARSH in government health facilities and corresponding “control” outpatient clinics (eg obstetrics, skin care) in government facilities	3 intervention sites in tertiary care hospitals located in medical colleges, all run outreach program in schools as well Each site evaluation included 4 staff member, 25 adolescent, and 25 parent interviews
**7** [42]	Maharashtra: Mumbai	2010	BY: Academic institution/university (National Institute for Research in Reproductive Health (NIRRH) and state government agency (Municipal Corporation of Greater Mumbai) FOR: Not specified	To test the feasibility of delivering ARSH services within public sector of Mumbai and to evaluate scaled up ARSH services at other health facilities	Delivering health services based on Government of India’s ARSH Programme	**(1) Feasibility assessment:** Observations on implementation of ARSH services within public sectors **(2) Quasi-experimental:** Pre- and post-scale up comparison of participants’ SRH knowledge help-seeking behaviours and time series comparison of health service utilization	(1) Focus group discussions with adolescents, teachers, parents, and other stakeholders (2) Pre- and post-scale up questionnaire (3) Monitoring of attendance data from clinic	Government primary care health posts with subsequent scale-up to include secondary care level hospitals	Research phase questionnaire participants *N* = 1326 adolescents interviewed of 1565 total adolescents using services at 2 health posts) Scale up phase questionnaire participants *N =* 2164 of 3250 adolescents using services at 3 health posts)
**8** [43]	Bihar: Nalanda, Nawada, Patna	2010	By: International NGO (Pathfinder International) FOR: International NGO (Pathfinder International)	To estimate the impact of implementing the PRACHAR model in the reproductive health and FP programs in Bihar and Uttar Pradesh	PRACHAR intervention (see above)	**Quasi-experimental:** Comparison of projected population growth between intervention and non-intervention communities	Population projection using computer programme SPECTRUM to evaluate change in two fertility parameters (total fertility rate and age-pattern of fertility) over period under projection 2005 and 2025	Type of health facilities within intervention not specified	Health facilities in intervention communities and number of adolescent participants using facilities not assessed
**9** [44]	Bihar: Gaya, Nalanda, Nawada, and Patna	2011	BY: International NGO (Pathfinder International) and consulting agency (India Institute of Health Management Research (IHMR)) FOR: Foreign government agency (USAID)	To conduct retrospective analysis of PRACHAR phase I and II data to develop a better understanding of the impact of FP/SRH outcomes and analyse possible trends in gender norms, attitudes, practices related to SRH that may have changed over time as result of PRACHAR -To conduct qualitative research (thru FGDs) to explore possible linkages between intended SRH behavioral outcomes, multisectoral elements (eg education) and PRACHAR’s gender accommodating and transformative elements and how gender inputs could be made stronger to enhance gender outcomes	PRACHAR intervention (see above)	**Multiple designs:** **(1) Quasi-experimental:** Comparison of attitudes and health behaviours between intervention community participants and comparison community participants **(2) Descriptive:** post-implementation qualitative study	(1) Post-implementation structured interview to assess history of marriage, reproductive health knowledge, attitudes, and behaviours, and pregnancy outcomes (2) Focus group discussions exploring gender and multisectoral factors, family relations and communication, education, work, support	Type of health facilities within intervention not specified	Health facilities in intervention communities and number of adolescent participants using facilities not assessed Evaluation sample = 23,400 intervention participants, 3900 baseline comparison participants, 7200 endline participants. Adolescent Follow-Up Study sample: 1224 participants who had been exposed to PRACHAR (306 M, 306 F, baseline and endline) 21 FGDs with 196 participants (varied from young women and men, mothers, fathers, community influences, trainers, field workers)
**10** [45]	Gujarat: Ahmedabad	2012	BY: Academic institution/university (Department of Community Medicine, Smt. NHL Municipal Medical College, Ahmedabad, Gujarat) FOR: Not specified	To evaluate knowledge regarding AFHS among Anganwadi workers (AWWs) To evaluate improvement in knowledge and skills of AWWs at appropriate intervals after skill-based training To assess health status and knowledge, attitudes, and practices (KAP) of adolescent girls of Anganwadis	Provision of a didactic education session with power point presentation, uterus model and chalkboard on importance of adolescent health to 111 AWWs in order to improve health services based on Government of India’s Adolescent Reproductive and Sexual Health Programme	**Multiple designs:** **Quasi-experimental:** Pre- and post-intervention comparison of knowledge of Anganwadi workers **Descriptive:** Cross-sectional assessment of health status and KAP of adolescent girls	Questionnaire measuring knowledge of Anganwadi workers Assessment tool to measure health status and KAP of adolescent girls not described	Type of health facilities within intervention not specified	Health facilities in intervention communities and number of adolescent participants using facilities not assessed Convenience sample of 111 AWWs for didactic education and questionnaire 142 adolescent girls were assessed for health status and KAP (target population unknown)
**11** [46]	**Bihar:** Nalanda, Nawada, Patna	2012	By: International NGO (Pathfinder International/Daniel et al) FOR: International NGO (Pathfinder International)	To assess the effect of intervention on age at marriage, contraceptive use before and after first birth, age at first birth	PRACHAR intervention (see above)	**Quasi-experimental:** post-implementation comparison of behavioural and health outcomes between participants in intervention communities versus those in non-intervention communities	Post-implementation structured interview using questionnaire to assess history of marriage, reproductive health knowledge, attitudes, and behaviours, and pregnancy outcomes	Type of health facilities within intervention not specified	Health facilities in intervention communities and number of adolescent participants using facilities not assessed
**12** [47]	**Uttar Pradesh:** Arajiline block of Varanasi district and Hosakote block of Bangalore district	2013	BY: Indigenous NGO (Research Unit at MAMTA-Health Institute for Mother and Child, Delhi) FOR: Not specified	To describe features of the intervention and to investigate (1) the impact on improving awareness and utilization of services by adolescents and (2) the quality of ARSH services in the intervention districts	Delivering health services based on Government of India’s ARSH Programme	**Descriptive:** post-implementation cross-sectional study of quality of services comparing two intervention districts	Community-based survey of adolescents Exit interviews questionnaire of adolescent clients -Structured facility questionnaire to measure staffing, training, infrastructure, supplies, and services -Measurement of health service utilization	Youth friendly health facilities not further specified	17/217 villages in Arajliine and 17/333 villages in Hosakote with 12 girls and 12 boys selected from each → total sample = 737 adolescents (383 M, 354 F) Consecutive sample of 120 clients from 4 selected clinics for exit interviews

**Table 5:** Main findings of evaluations (*N =* 18)

	Findings from the evaluations of:					
ID	Design	Implementation	Outputs (quality and coverage)	Health behaviour outcomes	Health outcomes	Comments
Eg.	Project goal Project objectives Framework of design Key approaches and strategies Rationale or basis Key actors	Implementation plan Activities undertaken Key influences Monitoring plan Use of monitoring information Mid-course adaptations/changes	Pre-inputs: Training material, training of trainers, clinical monitoring (CM), CM training, supportive supervision (SS) material, SS training Inputs: Training of health service providers, making facilities AFHS, problem solving, SS Outputs: Improved quality of services Outcomes: Improved use of services	Effect on adolescent behaviour (sexual behaviour, condom/contraceptive use behaviour/health seeking behaviour)	Eg. nutritional status, early pregnancy and pregnancy related mortality and morbidity, STIs and HIV	Other evaluation dimensions: community support and adolescent demand, planning and management, institutionalisation, cost
**A**				Better Life Options participants were more likely to have received antenatal care during pregnancy (91% vs. 64%), received tetanus toxoid immunization during pregnancy (91% vs. 62), delivered in health facility (50% vs. 36%), received post-natal care (57% vs. 39%), and currently be using contraception (36% vs. 27%) Children of participants 12 months or older more likely to have received complete primary immunizations (63% vs. 32%) Better Life Options participants were more likely to report having given child oral rehydration salts during diarrhea (42% vs. 12%)	Better Life Options participants had lower mean number of children (1.73 vs. 1.98) Better Life Options participants had lower rates of child deaths (RR = 0.88)	Other social outcomes including age at marriage, level of education completed, literacy were also were also evaluated
**B**		Intervention was feasible (focus on general adolescent health very effective; programme faced little resistance from parents, programme implementers, schools)		Statistically significant (*p* < 0.01) percent change (%Δ) in knowledge of modern methods of contraception (male/female sterilization 37.6%Δ, condoms 34.6%Δ. intrauterine device 25.0%Δ, desire for less than 3 children 13.1%Δ, knowledge about need for 3 ANC checkups%Δ Statistically significant (*p* < 0.01) increases in knowledge for each of 4 modes of HIV transmission (sharing needles 39.8%Δ, unprotected sex 30.1%Δ, maternal to child transmission 32.3%Δ, blood transfusion with infected blood 35.0)%Δ	Statistically significant (*p* < 0.01) reduction in proportion of participants with anemia (HGB < 10 grams) from 86% to 20% among 10-14 yo and 86% to 36% among 15-19 yo Mean Hgb level improved (9.0 grams/d: to 11.1 grams/dL in 10-14 yo, 9.0 grams/dL to 10.7 grams/dL in 15-19 yo)	
**C**		Evaluation identified program implementation, noting that ARSH-related supplies were found to be available, but not uniformly being distributed to adolescents	Difference in quality scores between ARSH and other clinics for each standard (statistical significance is not reported): 1. Health facilities provide the specific package of health services that adolescents need: 65% in ARSH vs. 22% in other 2. Health facilities deliver effective services to adolescents: 78% vs. 39% 3. Adolescents find the environment at health facilities conducive to seeking treatment: 86% vs. 33% 4. Service providers are sensitive to adolescent needs and are motivated to work with them: 94% vs. 59% 5. An enabling environment for adolescents to seek services exists in community: 63% vs. 12% 6. Adolescents are well informed about health services: 44% vs. 1% 7. Management systems are in place to improve/sustain the quality of health services: 45% vs. 13% No apparent difference in performances of PHCs vs. SCs Most intervention sites progressing well towards meeting the standards			
**D**			Awareness of AFHS 8x higher in intervention area than comparison villages (68% vs. 8%)° Use of government health facilities was higher in intervention than comparison villages (55% v.37%)° Denial of contraceptive services was perceived by majority of adolescents in BOTH intervention and comparison villages More adolescents in intervention villages understood explanations of health problems than in comparison (83% v. 42%)° No major differences between groups in acceptability and availability of condoms			
**E**		Some centres were non-functional due to transfer of MO who was oriented about centre and lack of human resources Major challenges to monitoring exist	Utilization data (average number of adolescent patients/month) showed minimal utilization. Where records available, average 250 adolescent clients/month. Focus groups revealed that adolescent boys and girls are generally unaware of ARSH centres and/or services. Use of services related to RSH problems is limited due to lack of awareness and knowledge			Quality of health services based on provider report, not direct observation, and scoring performed by evaluation team
**F**		Only 1 facility (SDH) was “designated” AFHS at time of assessment	Proportion of 7 standards of ASRH services implemented at each facility ranged from 19% to 42% Single facility that had been designated AFHS (SDH in Karjat) scored 31% Most broadly implemented standard (57% of facilities met standard) was standard 4: “Service providers are sensitive to adolescent needs and motivated to work with them.” Least implemented standard (1% of facilities met standard) was standard 6 (“Adolescents are well-informed about health services.”)			Positive feasibility of using quality assessment tools Comment that focus group discussions are needed to gather better inputs for standards V and VI
**G**		83% of clinics had been functional for less than one year	42% maintained audio and visual privacy 58% had displayed boards and 25% had adequate signage No ARSH facilities were found to have at least 100 condoms or at least 10 cycles of OCPs available, 83% had ECPs available 67% had access to ARSH guidelines Utilization of services low: 14.5% of adolescents interviewed had used clinic in past 6 months) Few providers felt adequately trained			
**H**			53,137 adolescents (40% male, 60% female) accessed services by 73 clinics -Scope of services included contraceptive choices, handling concerns related to menstruation and gender-based violence, improving life skills, providing antenatal services, treatment of Reproductive Tract Infections/Sexually Transmitted Infections Access and quality of services were quite limited (report did not provide data to support this)			
**I**			Adolescent health care available in 85.4% of SCs in 7 different states School health programs in 77% of PHCs and related facilities in different states ASHA participation in sensitizing adolescent girls was found to be unsatisfactory (Data not provided to support this)			
**J**				If comprehensive intervention is discontinued, there is an initial decline in contraceptive use in both groups after activities end, then stabilizes at higher level than pre-intervention Longer duration of comprehensive intervention was associated with greater increase in contraceptive use (a more modest effect demonstrated over shorter (2-3y) period of time Joint exposure of young married couples to PRACHAR communications is more effective than exposure to men alone PRACHAR interventions led to increased contraceptive use among all socioeconomic and education groups, but highest impact in most disadvantaged		
**K**			AWW and ASHA stakeholders have more knowledge about health concerns/programs meant for adolescents than did teacher and Panchayati Raj Institution members Quality of services most adequate in relation to facility measure (separate room, exam table, display boards, records/registers, weighing scale) and supply measures (condoms, Oral Contraceptive Pills, Emergency Contraception, etc.) IEC materials and outreach services, co-curricular education activities are lacking	Low SRH knowledge in adolescent community		
**L**		Scale-up included changes based on findings from evaluation of pilot intervention, including the addition of new service delivery points, shift in monitoring responsibilities, improvements in efficiency of services		Improved attitudes and behaviours related to reproductive and sexual health including decrease on preference for male child from 39.9% to 25.7% (*p* < 0.01), increased awareness of legal minimum age of marriage for girls from 68.2% to 85.4% (*p* < 0.01), and increased use of sanitary pads increased from 30.6% to 52.7% (*p* < 0.01)		
**M**			Majority (90%) of clients aware that YFHS provide services to young men and women separately on specific day/time 66% of clients visited YFHS to seek treatment for 3 key problems (menstruation, general illness, swelling/itching of genitals) Privacy not consistently ensured according to clients			
**O**			32% of interviewed clients reported satisfaction with AFHS Satisfaction was positively associated with female gender, higher education status, Hindu religion Multivariate model showed greater satisfaction associated with parental support (OR = 4.4), much lower satisfaction associated with fear of privacy disclosure to parents (OR = 0.08) – this factor was more important than parental attitudes Client satisfaction did not vary by appropriateness of time given by provider, clients’ belief regarding confidentiality of information, provision of information request			
**P**			Raw quality scores showed steady improvement with average score of 83% across all 8 health facilities in 5^th^ year of evaluation and 79% across 12 sub-centres. No statistical analysis of change in scores over time. Persistently low performance of standard II (effectiveness of health facilities, including equipment and supplies)			
**Q**	Situation analysis informed development of the following interventions as a block action plan: (1) Refresher trainings for providers (2) Linkages with schools ad community organizations (3) Standard operating procedures and management information systems (4) Demand generation through collaboration with education department (5) Mobile helpline service (6) Quality assessment program evaluation at the end of each year to be performed by external evaluators -	Feasibility assessment of block action plan found that: (1) Adolescents will not come to clinic on a particular ARSH day, so clinic schedule shifted to “anytime approach” in the block (2) Medical camps for adolescents helped strengthen linkages with schools/colleges, parents, and teachers and have facilitated demand generated (3) Linkages with NGOs helped created awareness of ARSH services (4) Proactive involvement of education system and clear guidelines are essential (5) Referrals within the block are not helpful since quality at sub-district hospitals are not superior(6) Interventions with limited scope were peer volunteers, mobile line service, and an adolescent health committee (7) Clear cut guidelines on ARSH exist from Government of India, but no departments except health sector have specific policies for roles and responsibilities related to adolescents	Findings of quality assessment program reported in separate evaluation document (43)			
**R**			Most health care providers had undergone some training or sensitisation on SRH issues relevant to adolescents – some within context of general training and others through special training programs ASHA and ANM training more often focused on “safer issues” like nutrition and menstrual hygiene while counsellors and MOs also received training on sexual relations, infection, pregnancy, and abortion. Fewer training experiences with privacy, confidentiality, non-judgemental interaction, promotion of informed choice, and communication skills “Cascade approach” to training reaches large numbers, but not successful in building capacity on intractable aspects of service provision like building communication skills and overcoming discomfort in talking about SRH issues Gendered responses regarding what information adolescents should receive, most believed that information provision to girls should be mothers and female providers like ASHAs and ANMS while boys should get information from other males (MOs, counsellors, etc) Providers generally observe that adolescents and youth do not access SRHCS available at community level or at AFHCs at facility levels Based on exit interviews and mystery clients, suitability of services was mixed with most commonly cited complaint being lack of privacy	<50% of surveyed men and <66% of surveyed women who experienced an SRH problem had sought advice and/or treatment, fewer (33%) for mental health concerns. Most sought treatment from medical officers in government or private facilities. Adolescents reported limited interaction with frontline and community HCPs (ANMs, ASHAs, and AWWs) Awareness of AFHCs was low among adolescents (5% of young men and 8% of young women surveyed were aware of services), <1% had ever sought services		Report also summarizes the perceived health problems among adolescents surveyed as well as their preferences about health care providers and facilities
**S**			Data on health facilities providing ARSH services is sparse and only covers public facilities. There has been insufficient training of providers of these services. Quality of services is poor due to lack of manpower, lack of trained manpower, space constraints, poor community participation, time constraints. Knowledge of medical care providers and majority of paramedical care providers was sufficient, however majority of paramedics reported lack of comfort in communicating with adolescent clients. Facility surveys reveal lack of optimum IEC/BCC material, inadequate space for privacy, and long patient queues. 77% of facilities had adequate stock of key supplies. Adolescent exit interviews reiterated above issues and also reported long waiting times, stigma of being seen in facility, inappropriate clinic hours/days, and low understanding by family and community members for SRH needs	Low awareness of ARHS problems and availability of service among adolescents in community		

References

17. Rashtriya Kishor Swasthya Kayrakram. Strategy Handbook. New Delhi. 2014.

18. The Centre for Development and Population Activities (CEDPA). (2001). *Adolescent Girls in India Choose a Better Future* *: An Impact Assessment*. Washington, DC. Retrieved from http://nipccd.nic.in/mch/fr/dom/erl1.pdf

19. Mishra, A., Levitt-Dayal, M., & The Centre for Development and Population Activities (CEDPA). (2003). *Improving Adolescent Reproductive Health Knowledge and Outcomes through NGO Youth-Friendly Services*. Washington, DC.

20. Society for Women and Children’s Health and Government of India/Ministry of Health and Family Welfare. (2008). *Coverage Survey on Adolescent Friendly Health Services in a District in Haryana*.

21. Government of India/Ministry of Health and Family Welfare. (2008). *Status of Adolescent Friendly Health Services in Haryana*.

22. Centre for Operations Research and Training. (2008). *Assessment of Adolescent Reproductive and Sexual Health (ARSH) Centers in Gujarat: A Report*. Vadodara, India. Retrieved from http://www.cortindia.in/RP%5CRP-2008-01.pdf

23. Government of India/Ministry of Health and Family Welfare. (2009). Status of Adolescent Friendly Health Services in Karjat Block, Maharashtra.

24. Institute of Health Management Research. (n.d.). *Assessment of Adolescent Friendly Health Clinics in Rajasthan*. Jaipur, India.

25. Nanda, A., Mehrotra, F., Verma, R., Nanda, P., & Masilamani, R. (2011). Evaluation Report of UNFPA India Country Programme-7 UNFPA Country Office, India Evaluation Team.

26 Programme Evaluation Organisation Planning Commission. (2011). *Evaluation Study on National Rural Health Mission (NRHM) in Seven States: Volume II*. New Delhi.

27. Pathfinder International. (2011). *PRACHAR: Promoting Change In Reproductive Behavior In Bihar, India*.

28. Bulliyya, G., & Kerketta, A. S. (2012). *Assessment of adolescent reproductive and sexual health programme in Orissa: advocacy for intervention strategies*.

29. Futures Group. (2012). *Promoting adolescent reproductive health in Uttarakhand and Uttar Pradesh, India*. Retrieved from http://futuresgroup.com/files/publications/Adolescent.pdf

30. Mehra, S., Sogarwal, R., Nair, V., Satpati, M., Tiwari, R., & Dwivedi, K. (2013). Determinants of Youth Friendly Services Influencing Client Satisfaction: A Study of Client’s Perspectives in India. *Indian Journal of Public Health Research & Development*, *4*(2), 221. http://doi.org/10.5958/j.0976-5506.4.2.047

31. Mehra, S., Sogarwal, R., & Chandra, M. (2013). Integrating adolescent-friendly health services into the public health system: an experience from rural India. *WHO South-East Asia Journal of Public Health*, *2*(1), 6. http://doi.org/10.4103/2224-3151.115828

32. National Institute for Research in Reproductive Health (Indian Council of Medical Research). (2014). *Quality Assessment of Adolescent Reproductive and Sexual Health (ARSH) Services as per the National Standards of ARSH Implementation Guide in Karjat Block of Raigad District in Maharashtra, India*.

33. National Institute for Research in Reproductive Health (Indian Council of Medical Research). (2014). Establishing, Operating, Strengthening and SUstaining the Adolescent Reproductive and Sexual Health (ARSH) Services in Karjat Block of Raigad District in Maharashtra, India.

34. Jejeebhoy, S., Santhya, K., Singh, S., Rampal, S., Saxena, K., & Population Council. (2014). *Provision of adolescent reproductive and sexual health services in India* *: Provider perspectives*.

35. Sharma, P., Ingle, G., Kamra, S., & Agarwal, P. (2014). *A Study of Gaps in Reproductive Health Services for Adolescents in Delhi*. New Delhi.

36. International Center for Research on Women. (2006). Improving the Reproductive Health of Married and Unmarried Youth in India: Reproductive and Sexual Health Education, Care and Counseling for Married Adolescents in Rural Maharashtra, India. Retrieved from http://www.icrw.org/files/images/Reproductive-and-Sexual-Health-Education-Care-and-Counseling-for-Married-Adolescents-in -Rural-Maharashtra-India.pdf

37. Joshi, B. N., Chauhan, S. L., Donde, U. M., Tryambake, V. H., Gaikwad, N. S., & Bhadoria, V. (2006). Reproductive health problems and help seeking behavior among adolescents in urban India. *Indian Journal of Pediatrics*, *73*(6), 509–13. Retrieved from http://www.ncbi.nlm.nih.gov/pubmed/16816513

38. International Center for Research on Women. (2006). *Improving the Reproductive Health of Married and Unmarried Youth in India: Social Mobilization or Government Services: What Influences Married Adolescents’ Reproductive Health in Rural Maharashtra, India?* Retrieved from http://www.icrw.org/files/images/Social-Mobilization-or-Government-Services-What-Influences-Married-Adolescents-Reproductive-Health-in-Rural-Maharashtra-India.pdf

39. Society for Women and Children’s Health and Government of India/Ministry of Health and Family Welfare. (2008). *Coverage Survey on Adolescent Friendly Health Services in a District in Haryana*.

40. Daniel, E. E., Masilamani, R., & Rahman, M. (2008). The effect of community-based reproductive health communication interventions on contraceptive use among young married couples in Bihar, India. *International Family Planning Perspectives*, *34*(4), 189–97. http://doi.org/10.1363/ifpp.34.189.08

41. Yadav, R. J., Mehta, R., Pandey, A., & Adhikari, T. (2009). Evaluation of Adolescent-Friendly Health Services in India. *Health and Population: Perspectives and Issues*, *32*(2), 66–72.

42. Chauhan, S., Joshi, B., Bandiwadekar, A., Barathe, U., Tryambake, V., & Gaikwad, N. (n.d.). *Scaling-up adolescent friendly health services within public sector in Mumbai, India*.

43. Rahman, M., & Daniel, E. E. (2010). *A Reproductive Health Communication Model That Helps Improve Young Women’s Reproductive Life and Reduce Population Growth: The Case of PRACHAR from Bihar, India*. Retrieved from https://researcharchive.lincoln.ac.nz/bitstream/10182/1720/3/institutional_barriers.pdf.txt Pathfinder International. (2011).

44. PRAGYA: Multisectoral, Gendered Approach to Improve Family Planning and Sexual and Reproductive Health for Young People: A Research Study, (December). Retrieved from http://www.pathfinder.org/publications-tools/pdfs/PRAGYA_Multisectoral_Gendered_Approach_to_Improve_Family_Planning_and_Sexual_and_Reproductive_Health_for_Young_People.pdf

45. Chauhan, S. R., Dalal, A. P., & Shukla, A. A. (2012). INTERVENTIONAL STUDY TO STRENGTHEN THE “ADOLESCENT FRIENDLY HEALTH SERVICES” IN ANGANWADIS OF AHMEDABAD MUNICIPAL, *3*(4), 617–622.

46, Daniel, E. E., & Nanda, R. (2012). *The Effective of Reproductive Health Communication Interventions on Age at Marriage and First Birth in Rural Bihar, India: A retrospective study*.

47. Mehra, S., Sogarwal, R., & Chandra, M. (2013). Integrating adolescent-friendly health services into the public health system: an experience from rural India. *WHO South-East Asia Journal of Public Health*, *2*(1), 6. http://doi.org/10.4103/2224-3151.115828

48. Andrew, G., Patel, V., & Ramakrishna, J. (2003). Sex, studies or strife? What to integrate in adolescent health services. *Reproductive Health Matters*. http://doi.org/10.1016/S0968-8080(03)02167-0

49. Biswas, R. (2002). An overview of multicentric training workshops for public health professionals on reproductive and child health programme in India. *Indian Journal of Public Health*, *46*(3), 78–85.

50. Calhoun, L. M., Speizer, I. S., Rimal, R., Sripad, P., Chatterjee, N., Achyut, P., & Nanda, P. (2013). Provider imposed restrictions to clients’ access to family planning in urban Uttar Pradesh, India: a mixed methods study. *BMC Health Services Research*, *13*, 532. http://doi.org/10.1186/1472-6963-13-532

51. Char, A., Saavala, M., & Kulmala, T. (2011). Assessing young unmarried men’s access to reproductive health information and services in rural India. *BMC Public Health*, *11*(1), 476. http://doi.org/10.1186/1471-2458-11-476

52. Char, A., Saavala, M., & Kulmala, T. (2010). Influence of mothers-in-law on young couples’ family planning decisions in rural India. *Reproductive Health Matters*, *18*(35), 154–62. http://doi.org/10.1016/S0968-8080(10)35497-8

53. Collumbien, M., Mishra, M., & Blackmore, C. (2011). Youth-friendly services in two rural districts of West Bengal and Jharkhand, India: Definite progress, a long way to go. *Reproductive Health Matters*, *19*, 174–183. http://doi.org/10.1016/S0968-8080(11)37557-X

54. Das, D. (2006). Morbidity and treatment seeking behavior among adolescent girls in a rural area of North 24 Parganas district, West Bengal. *Indian Journal of Public Health*, *50*(4), 242–243.

55. De Souza, R. (2014). A Qualitative Study of Roles Performed by Peer Workers in the Context of HIV in India. *Journal of the Association of Nurses in AIDS Care*, *25*, 176–187. http://doi.org/10.1016/j.jana.2013.01.004

56. Dongre, A. R., Deshmukh, P. R., & Garg, B. S. (2011). Health-promoting school initiative in Ashram schools of Wardha district. *The National Medical Journal of India*, *24*(3), 140–3. Retrieved from http://www.ncbi.nlm.nih.gov/pubmed/21786841

57. Hazarika, I. (2010). Women’s reproductive health in slum populations in India: evidence from NFHS-3. *Journal of Urban Health* *: Bulletin of the New York Academy of Medicine*, *87*(2), 264–77. http://doi.org/10.1007/s11524-009-9421-0

58. Kotecha, P. V, Patel, S. V, Mazumdar, V. S., Baxi, R. K., Misra, S., Diwanji, M., … Shringarpure, K. (2012). Reproductive health awareness among urban school going adolescents in Vadodara city. *Indian Journal of Psychiatry*, *54*(4), 344–8. http://doi.org/10.4103/0019-5545.104821

59. Mishra, S. K., & Mukhopadhyay, S. (2012). Socioeconomic correlates of reproductive morbidity among adolescent girls in Sikkim, India. *Asia-Pacific Journal of Public Health/Asia-Pacific Academic Consortium for Public Health*, *24*(1), 136–50. http://doi.org/10.1177/1010539510375842

60. Nair, M. K. C., Leena, M. L., Thankachi, Y., George, B., & Russell, P. S. S. (2013). ARSH 1: Reproductive and Sexual Health Problems of Adolescents and Young Adults: A Cross Sectional Community Survey on Knowledge, Attitude and Practice. *Indian Journal of Pediatrics*, pp. 1–7. http://doi.org/10.1007/s12098-013-1136-2

61. Nair, M. K. C., Leena, M. L., George, B., Thankachi, Y., & Russell, P. S. S. (2013). ARSH 5: Reproductive Health Needs Assessment of Adolescents and Young People (15-24 y): A Qualitative Study on “Perceptions of Community Stakeholders.” *Indian Journal of Pediatrics*, pp. 1–8. http://doi.org/10.1007/s12098-013-1141-5

62. Nair, M. K. C., Leena, M. L., George, B., Thankachi, Y., & Russell, P. S. S. (2013). ARSH 6: Reproductive Health Needs Assessment of Adolescents and Young People (15-24 y): A Qualitative Study on “Perceptions of Program Managers and Health Providers.” *Indian Journal of Pediatrics*, pp. 1–7. http://doi.org/10.1007/s12098-013-1149-x

63. Nair, M. K. C., Leena, M. L., Paul, M. K., Vijayan Pillai, H., Babu, G., Russell, P. S., & Thankachi, Y. (2012). Attitude of parents and teachers towards adolescent reproductive and sexual health education. In *Indian Journal of Pediatrics* (Vol. 79). http://doi.org/10.1007/s12098-011-0436-7

64. Nath, A., & Garg, S. (2008). Adolescent friendly health services in India: A need of the hour. *Indian Journal of Medical Sciences*, *62*, 465–472. http://doi.org/10.4103/0019-5359.48461

65. Rao, R. S. P., Lena, A., Nair, N. S., Kamath, V., & Kamath, A. (2008). Effectiveness of reproductive health education among rural adolescent girls: a school based intervention study in Udupi Taluk, Karnataka. *Indian Journal of Medical Sciences*, *62*, 439–443. http://doi.org/10.4103/0019-5359.48455

66. Sabarwal, S., & Santhya, K. G. (2012). Treatment-seeking for symptoms of reproductive tract infections among young women in India. *International Perspectives on Sexual and Reproductive Health*, *38*(2), 90–8. http://doi.org/10.1363/3809012

67. Shah, S. P., Nair, R., Shah, P. P., Modi, D. K., Desai, S. a, & Desai, L. (2013). Improving quality of life with new menstrual hygiene practices among adolescent tribal girls in rural Gujarat, India. *Reproductive Health Matters*, *21*(41), 205–13. http://doi.org/10.1016/S0968-8080(13)41691-9

68. Sharma KD, Chavan YB, Khismatrao DS, Aras RY. Male health clinic strategy in control of STI/HIV: A program review. Indian J Public Health. 2012;56:238–41.

69. Singh, L., Rai, R. K., & Singh, P. K. (2012). Assessing the utilization of maternal and child health care among married adolescent women: evidence from India. *Journal of Biosocial Science*, *44*(1), 1–26. http://doi.org/10.1017/S0021932011000472

70. Speizer, I. S., Nanda, P., Achyut, P., Pillai, G., & Guilkey, D. K. (2012). Family planning use among urban poor women from six cities of Uttar Pradesh, India. *Journal of Urban Health* *: Bulletin of the New York Academy of Medicine*, *89*(4), 639–58. http://doi.org/10.1007/s11524-011-9667-1
